# Brain functional connectivity analysis of fMRI-based Alzheimer's disease data

**DOI:** 10.3389/fmed.2025.1540297

**Published:** 2025-02-19

**Authors:** Maitha S. Alarjani, Badar A. Almarri

**Affiliations:** Computer Science Department, College of Computer Sciences and Information Technology (CCSIT), King Faisal University, Al-Ahsa, Saudi Arabia

**Keywords:** Alzheimer's disease, cognitive, functional connectivity, extreme learning machine, machine learning, computational analysis

## Abstract

The prevalence of Alzheimer's disease (AD) poses a significant public health challenge. Distinguishing AD stages remains a complex process due to ambiguous variability within and across AD stages. Manual classification of such multifaceted and massive data of brain volumes is operationally inefficient and vulnerable to human errors. Here, we propose a precise and systematic framework for AD stages classification. The core of this framework discovers and analyzes functional connectivity among regions of interest (ROIs) of a human brain. Multivariate Pattern Analysis (MVPA) is applied to extract features that reveal complex functional connectivity patterns in the brain. These features are then used as inputs for an Extreme Learning Machine (ELM) model to classify AD stages. The model's performance is assessed through comprehensive evaluation metrics to ensure robustness and reliability. Applying this framework on datasets which contain meticulously validated fMRI scans such as the OASIS and AD Neuroimaging Initiative datasets, we validate the merit of this proposed work. The framework's results show improvement in the collective performance of two-class and multi-class classification. Feeding ELM with MVPA features yield decent outcomes given a generalizable and computationally-efficient model. This study underscores the effectiveness of the proposed approach in accurately distinguishing AD stages, offering potential improvements in AD and AD stages detection.

## 1 Introduction

Alzheimer's disease (AD) is a progressive and irreversible brain disorder that slowly destroys memory and thinking capabilities, ultimately causing loss of cognitive function. It is one of the diseases that cause brain damage (1). The disease typically begins with mild memory loss and may be mistaken for normal aging. As it progresses, individuals may experience significant cognitive impairment, difficulty with language, problem-solving challenges, and a loss of the ability to carry out daily activities (2)

The buildup of tau tangles and amyloid-beta (A) protein in the brain are two of the main symptoms of AD. This neurodegenerative brain ailment progressively impairs cognition in its initial stages and eventually results in mortality. AD is a progressive condition that worsens over time. There are two subtypes of AD (3):

- Early-onset AD and late-onset AD Uncommon genetic abnormalities characterize early-onset AD in patients under the age of 65 (5%).
- AD with a late onset, which affects people with an age onset older than 65 years old (95%) and is not caused by inherited mutations.

**Figure 1:**
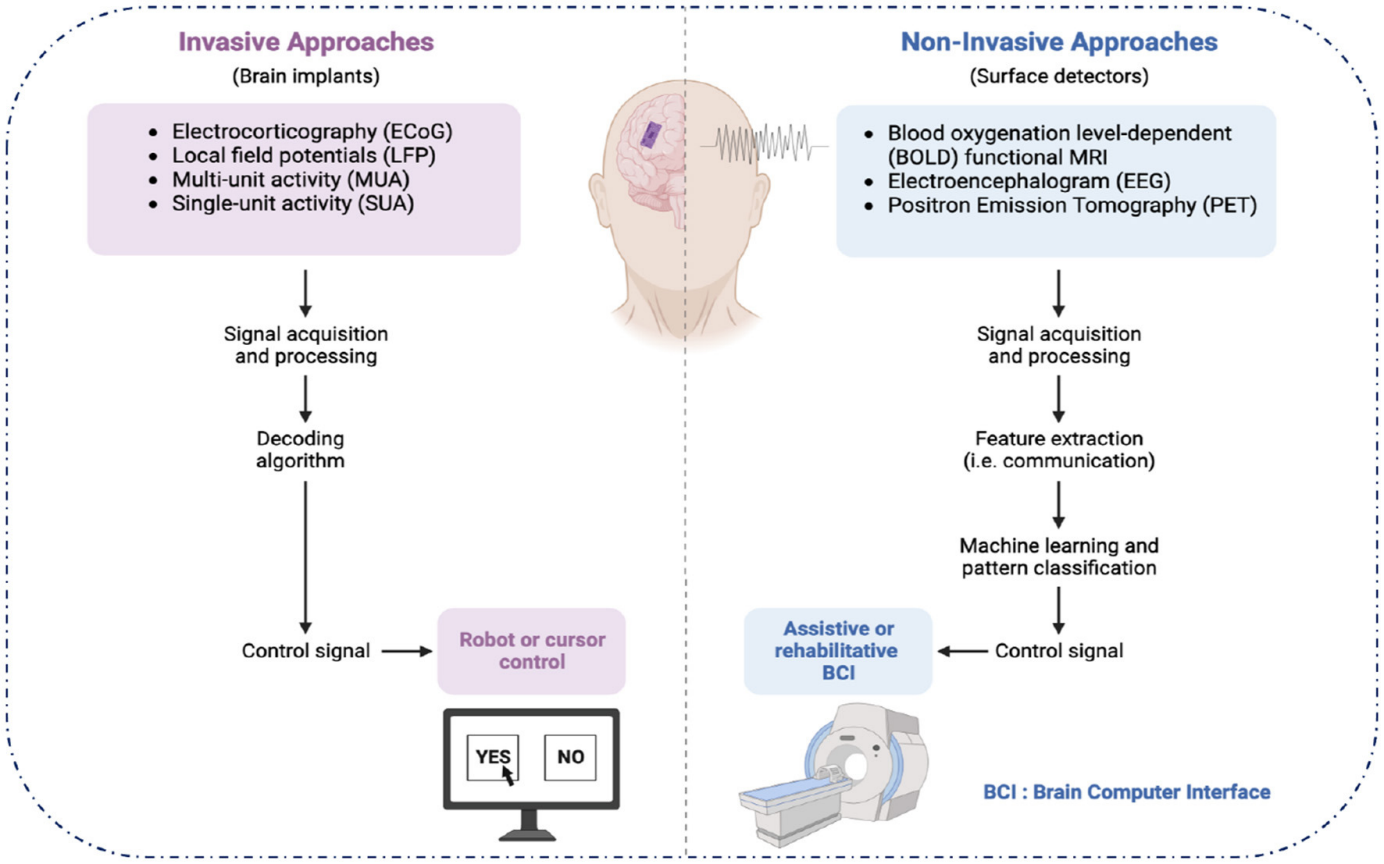
Invasive and non-invasive approaches.

Advanced neuroimaging techniques, including magnetic resonance imaging (MRI) and positron emission tomography (PET), allow researchers to detect structural and molecular markers of AD in a non-invasive way, offering both structural and functional insights without the need for physical intervention. Meanwhile, methods like cerebrospinal fluid (CSF) analysis and blood biomarker assessment, though invasive, provide crucial molecular information that can aid in early diagnosis and tracking of AD progression. As neuroimaging technologies continue to evolve at a rapid pace, the volume and complexity of multi-modal data generated are also increasing, posing challenges for data integration, interpretation, and management (4) (see Figure 1).

AD cannot be cured at this time. Some medications can be helpful in symptom control; however, these treatments cannot stop the course of the disease. Unfortunately, the experiments that were conducted to test various strategies, Drugs that diminish the amount of the A peptide or encourage its clearance via active or passive vaccination, for example, failed to show therapeutic efficacy. There have been several tried-and-true methods, such as medications that reduce the amount of A protein or encourage the elimination of the through active or passive vaccination (5, 6).

To address these challenges, computer-aided machine learning techniques have become essential in processing and analyzing neuroimaging data efficiently and accurately. Such techniques enable the extraction of subtle patterns and predictive markers from large datasets, which may otherwise be undetectable to the human eye. Early identification of AD and suitable treatment intervention are crucial aspects of care, as the illness can start years or even decades before dementia develops. As a result, there is a growing focus being placed on carrying out clinical tests in groups consisting of people with no persons with mild cognitive impairment (MCI) or dementia who are in danger of acquiring AD.

### 1.1 Research contributions

This recent systematic review highlighted the limited number of studies employing MVPA in Alzheimer's research (7). The scarcity was attributed to the complexity and technical challenges of MVPA, including the need for specialized knowledge and difficulties in data preprocessing, feature selection, and classification. Despite the numerous algorithms in the literature demonstrating high accuracy, our focus on MVPA is justified by its unique capability to compute the connectivity between brain regions, offering insights into the underlying patterns of neural activity that other methods might overlook. Our approach aims to enhance the understanding of AD by leveraging the advanced analytical power of MVPA, which could complement and potentially surpass existing methods in capturing the intricate network interactions in the brain.

The key focus areas of this research include:

- Understanding AD through brain connectivity patterns.
- Mapping fMRI scans to a parcellation model using the AAL3 brain atlas to investigate brain connectivity patterns using Machine learning.
- Defining a framework model using Multivariate Pattern Analysis (MVPA) for classification using ELM classifier.
- Evaluate the model using performance metrics by comparing with OASIS and ADNI datasets.

The remaining papers are arranged as follows: offers a comprehensive review of the literature on AD in Section 2, Section 3 The Theoretical Background of AD and brain imaging are explained, Section 4 details our methodology, including Data Information, Preprocessing Pipeline, Functional Connectivity, and Classification Algorithms for different stages. Section 5 presents the discussion, while Section 6 covers the conclusions and future directions.

## 2 Previous studies

Because early illness identification is so important, numerous scientists are interested in returning to this field to find a therapy for AD. Thus, the most essential works in this field will be presented in this part.

The Graph Convolutional Network (GCN) model, which was established in this study (8), has two main functions: it can be used to diagnose MCI by differentiating MCI from normal controls (NCs), and it can also be used to predict the risk of dementia by categorizing NCs from participants in the OASIS-3 dataset. Possibly having MCI but not yet receiving a formal diagnosis. The findings show that the recommended GCN outperforms both baseline GCN and Support Vector Machine (SVM), with a peak accuracy of 91.2% and an average accuracy of 80.3%, which is 23.5% higher than SVM and 11.7% higher than baseline GCN. Although individual FC often outperforms global FC, under the GCN design, global graphs with the right connections can occasionally achieve similar or even greater performance than individual graphs. GCN categorization is influenced by brain network connections like the default mode, visual, ventral attention, and somatomotor networks. In this study, Alarjani (9) identified key features in fMRI data using a patch-based approach (PBA) and two 3D-CNNs with fully connected layers. We applied decision tree and k-nearest neighbor classifiers to detect AD, MCI, and NC using the ADNI dataset, achieving improved classification performance compared to previous studies.

Kolla et al. (10) used data-driven analysis of resting-state fMRI data to examine functional brain nodes and their effects on graph metrics. It introduces a new measure known as “node-metric coupling” (NMC), which considers the connection between graph metrics and node sizes. The findings suggest that NMC may serve as a valuable biomarker for brain disorders, underscoring the critical need to account for variations in node sizes in neuroimaging studies.

In order to improve the diagnosis of moderate cognitive impairment (MCI), HE et al. (11) employed a graph convolutional neural network to extract information from functional brain networks automatically. GCN with multimodal connectivity, integrates diverse connectivity patterns, achieving 92.2% accuracy and an AUC of 0.988 on the ADNI dataset, outperforming previous methods.

Ĩsmail et al. (12) proposed a novel model combining Capsule Network (CapsNet) and Recurrent Neural Network (RNN) to capture the full 4D information of fMRI data for AD diagnosis. The trial's results demonstrate its effectiveness. Compared to normal control (NC), AD was identified with 94.5% accuracy, while 61.8% accuracy separated early eMCI from late moderate cognitive impairment (lMCI).

This study used the edge time series paradigm in network neuroscience to examine the connections between brain and behavior in AD and related diseases. An examination of data from 152 persons examined the connections between neuropsychological processes and functional brain networks. New connections between attention, limbic, front parietal, and default mode networks and linguistic, executive, cognitive, and attention processes were found by the study. These results demonstrate how edge time-series analysis can go beyond conventional functional connectivity techniques to reveal brain dynamics associated to illness (13).

Kadhim et al. (14) created a computer-aided brain diagnostic (CABD) system capable of detecting AD from brain scans. By employing feature extraction techniques along with fMRI and Positron-Emission Tomography methods, the AD Neuroimaging Initiative (ADNI) data is analyzed. The developed method demonstrates an impressive classification accuracy rate of 97.7% for ADNI images.

Amini et al. (15) developed a machine learning (ML) method for AD diagnosis. To predict the severity of the disorder, they used fMRI data and MMSE scores using CNN, Decision Trees, SVM, KNN, RF, and LDA. Their CNN approach achieved an efficiency of 96.7% and sensitivity rates of 98.1% for low, 95.2% for mild, 89.0% for moderate, and 87.5% for severe AD. These results indicate superior performance compared to previous techniques in detecting AD severity.

Wang et al. (16) created a classification system for fMRI data that differentiates between AD, early and late moderate cognitive impairment (EMCI and LMCI), and healthy controls (HC) using graph theory and machine learning. A dual graph theoretical approach was merged into a unique multi-feature selection technique. Classification accuracy varied from 83.30% to 96.80%, suggesting that graph and ML combined might be useful for diagnosing AD, particularly when local metrics are used to indicate functional changes in specific brain areas. This study explored deep learning for early Alzheimer's detection, focusing on fMRI data after finding genetic factors had little impact. A 3D CNN model achieved 92.8% accuracy using ADNI data, highlighting the potential of deep learning in medical imaging for AD prediction (17).

Alarjani and Almarri (18) applied multivariate pattern analysis (MVPA) to compute connectivity among nodes and to analyze activity across multiple brain voxels. The proposed approach is applied to two public datasets and then classified using various ML classifiers. The results were assessed using performance metrics, and comparisons were conducted to differentiate between various stages of AD.

Table 1 presents a comparative analysis of various studies on AD detection techniques, highlighting their respective methods and limitations. This assessment serves as a valuable reference for understanding the unique attributes and challenges associated with both the graph-based optimization approaches in the context of analyzing brain signals for AD detection.

**Table 1:** Comparison with existing studies.

**Research study**	**Techniques used**	**Advantages**	**Limitations**	
(8)	Graph	GCNs capture brain region relationships	Training GCNs on fMRI data requires
	Convolutional	and detection functional connectivity changes,	substantial computational resources
	Networks	improving early dementia detection	due to model complexity.
(9)	3D-CNNs extracted features,	Used two 3D-CNNs with dual input	Deep learning was applied solely
	and decision tree/k-NN classified	layers for enhanced feature extraction	for feature extraction, not for classification
(10)	ICA on fMRI data extracts FNC	NMC is used as a potential biomarker,	Results depend on chosen atlas,
	matrices and analyzes brain	revealing a new link between	affecting generalizability
	connectivity via graph metrics	node size and graph metrics	
(16)	Integrated graph measures with	Highlighted local brain measures'	Multi-feature selection and graph analysis
	ML for fMRI analysis	role in detecting cognitive impairments	are computationally intensive
(14)	CNNs combined with Histogram Feature	HFE and Canny edge	Performance depends on ADNI
	Extraction were used to classify AD and NC	detection improve	dataset quality and size,
	subjects by extracting gradient orientation features	feature detection	limiting generalizability
(13)	The Edge Time-Series method is used to investigate	Identifies dynamic interactions	A limited dataset size
	interrelationships between brain function	between various brain nodes	was used.
	and neuropsychological measures		
(11)	GCN extracts functional	Multimodal connectivity improves	The study included only
	brain network features	performance by combining patterns	NC and MCI groups
(12)	FC matrices were used for	Combined CapsNet and RNN for enhanced	Performance was lower in classifying
	feature extraction from fMRI data	spatial and temporal feature learning	lMCI vs. eMCI, with 61.8% accuracy
(15)	KNN, SVM, DT, LDA,	An effectively classifies Alzheimer's	Small datasets may lead to
	and RF were used to classify AD	stages using fMRI and MMSE scores	overfitting in some methods
(18)	MVPA analyzes brain connectivity	The study utilized the OASIS	The study lacks deep
	and voxel activity patterns	and ADNI datasets	learning models

## 3 Background

**Figure 2:**
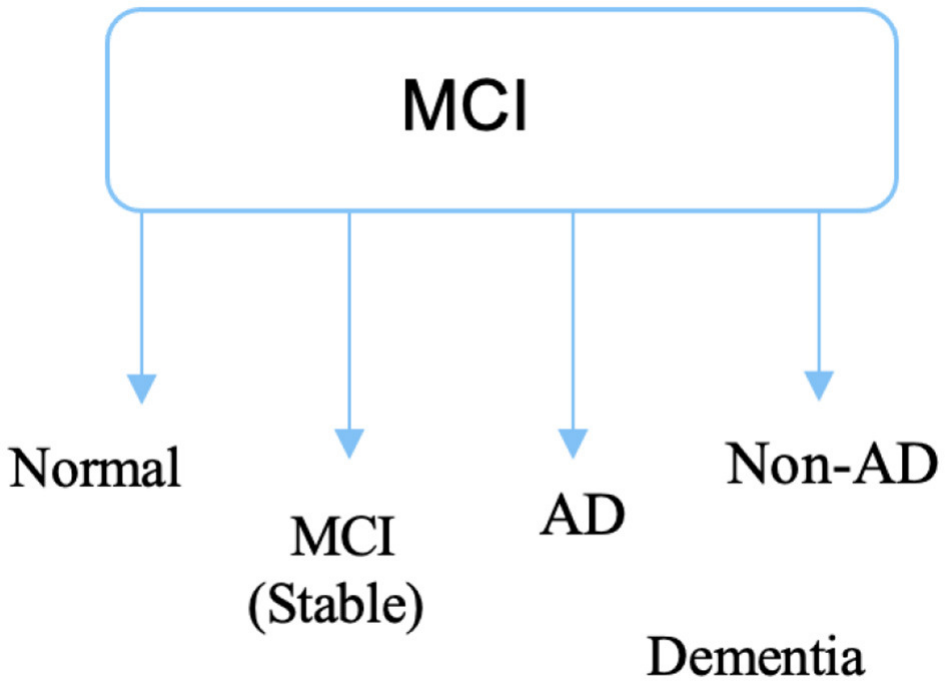
Phase of MCI stage.

AD progresses through stages: normal, very mild cognitive impairments, mild cognitive impairments, moderate dementia, and severe dementia (see Figure 2). MCI stages are mentioned among dementia and cognitively healthy people. Although MCI patients have lower cognitive function than healthy controls, their indications are less severe than those of Alzheimer's. There is a distinct deterioration in the association between the period with symptoms, MCI, and dementia phases of AD and typical cognitive aging. When someone has MCI, their thinking abilities show minor but significant changes that are visible to them and their family and friends, yet they are still capable of going about their daily lives. MCI affects about (15–20)% of persons 65 and older (19). A sizeable fraction of people can return to their normal cognitive process, whereas others can stay stable and avoid developing AD. The likelihood of acquiring AD is higher in people with antipsychotics and multi-domain MCI, while patients without dementia MCI are more likely to get other kinds of dementia (20, 21).

Brain imaging represents a transformative advancement in cognitive neuroscience. Before the advent of imaging technologies, brain research relied heavily on animal models and post-injury evaluations in humans. Diagnosing brain injuries posed significant challenges due to limited tools for assessing internal damage. Modern imaging techniques now provide precise methods for studying the brain, including mapping brain pathways, assessing brain density, and analyzing brain function (19).

Functional magnetic resonance imaging (fMRI) is among the most commonly used techniques for studying brain function, as it can capture high-resolution, real-time images of neural activity. This capability makes it invaluable for understanding both normal and abnormal brain processes. By detecting fluctuations in blood flow and oxygenation levels associated with neuronal activation, fMRI enables researchers to map specific regions of the brain that are engaged during various cognitive, sensory, and emotional tasks. This method provides insights into normal brain function, as well as potential abnormalities linked to neurological and psychiatric conditions. The depth of information fMRI offers makes it invaluable for studying complex neural networks, identifying biomarkers, and tracking subtle changes in brain function over time (22).

This work's primary focus is on computer-aided diagnostics. Achieving accuracy and reliability is crucial for its proper functioning in a healthcare setting, making it one of the most challenging issues in medical image processing. The goal is to categorize patients' cognitive states as CN, MCI, and AD based on their fMRI data. The categorization for AD diagnosis has been the subject of numerous investigations. In additional, He et al. (23) proposed the Spatiotemporal Graph Transformer Network (STGTN) for diagnosing AD using rs-fMRI data. Unlike traditional methods, STGTN captured spatiotemporal features and used adversarial training to enhance robustness and address limited sample sizes. The model achieved high accuracy (92.58% for AD vs. NC and 85.27% for eMCI vs. lMCI), outperforming existing methods. Additionally, it highlighted crucial brain connections for different classification tasks.

Support Vector Machine (SVM) was utilized by Jo et al. (24) to provide an enhanced AD classification system based on MRI indicators for categorizing CN vs. AD SVM was also utilized in Coupé et al. (25) to create a model that relied on the correlation of changes in transverse brain structure—multimodal indicators of MCI development with numerous kernel learning (KCL). However, use a model structure with k-Nearest Neighbors (KNN) (26). Suggested a best-resolution patch-based attributes extraction to retrieve cross-sectional and transverse information from MRI scans of the brain. Several medical fields have used deep learning, including image retrieval, segmentation techniques, image identification, and computer-aided diagnostics.

## 4 Methodology

**Figure 3:**
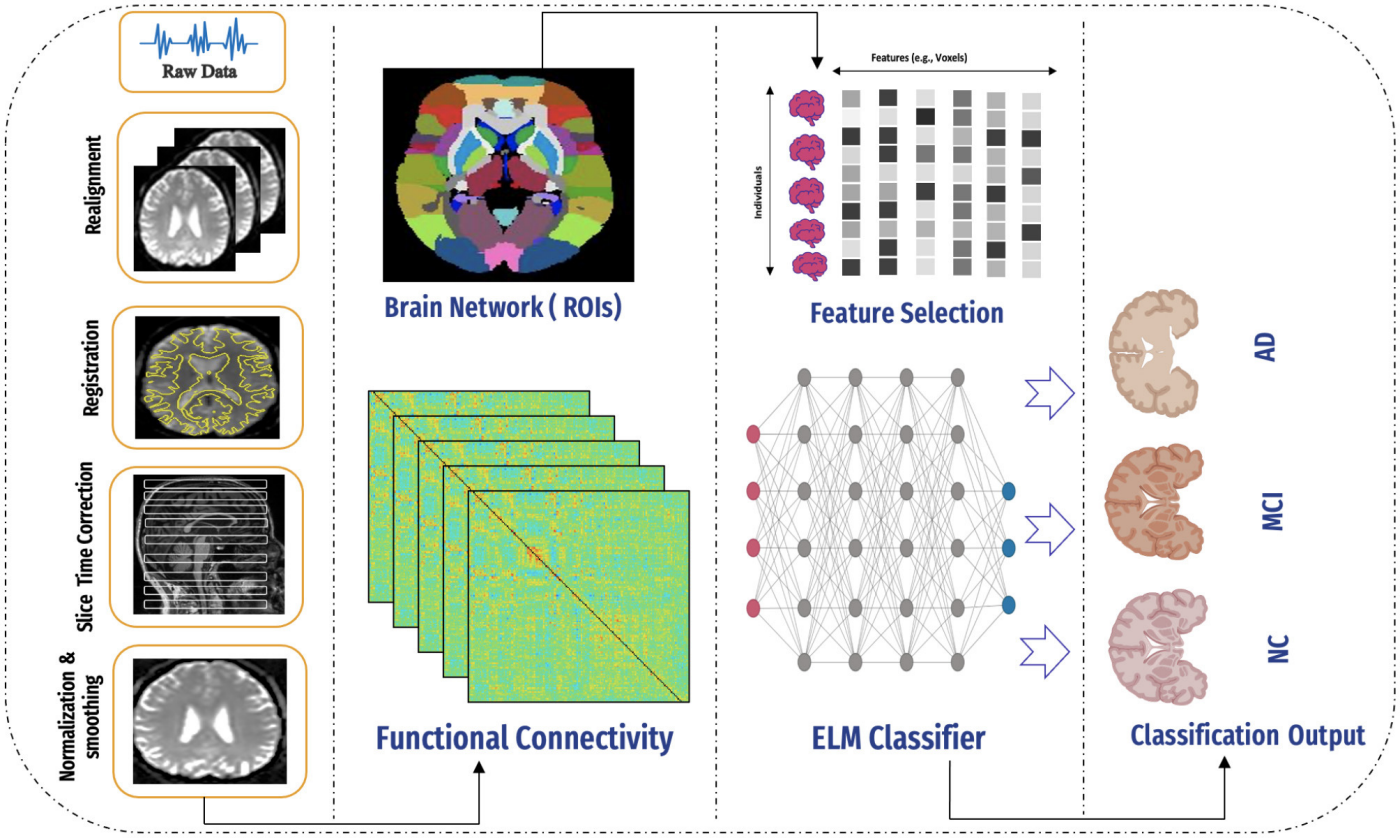
An overview of the proposed framework, including data collection, preprocessing, application of the AAL atlas, connectivity computation, feature selection, model application, and final classification using the ELM classifier.

The proposed system framework, depicted in Figure 3, consists of several key stages: data collection, data preprocessing, feature engineering, functional connectivity analysis, classification, results generation, and analysis. Each stage is integral to the system's workflow, facilitating the precise and efficient processing of data. The subsequent sections provide a detailed overview of each stage and its role in the framework.

### 4.1 Dataset collection and preparation

This study leverages two extensively used neuroimaging databases, the AD Neuroimaging Initiative (ADNI) and the Open Access Series of Imaging Studies (OASIS), which are critical resources in Alzheimer's research. These datasets provide a comprehensive range of imaging and clinical data, enabling detailed analyses of disease progression, early detection, and biomarker discovery.

The rs-fMRI dataset from ADNI was selected for its ability to capture brain activity during resting states without external stimuli. The dataset includes both male and female participants in different stages of AD (27). Specifically, 55 individuals (30 males and 25 females) were selected for the AD stage, 35 individuals (20 males and 15 females) for MCI, and 95 individuals (50 males and 45 females) for NC. Imaging data were collected using a Philips scanner with an 80° flip angle. To address the class imbalance inherent in the dataset, the Synthetic Minority Oversampling Technique (SMOTE) was employed. SMOTE generates synthetic examples for underrepresented classes, effectively balancing the data set and enhancing the performance of the model (28).

**Figure 4:**
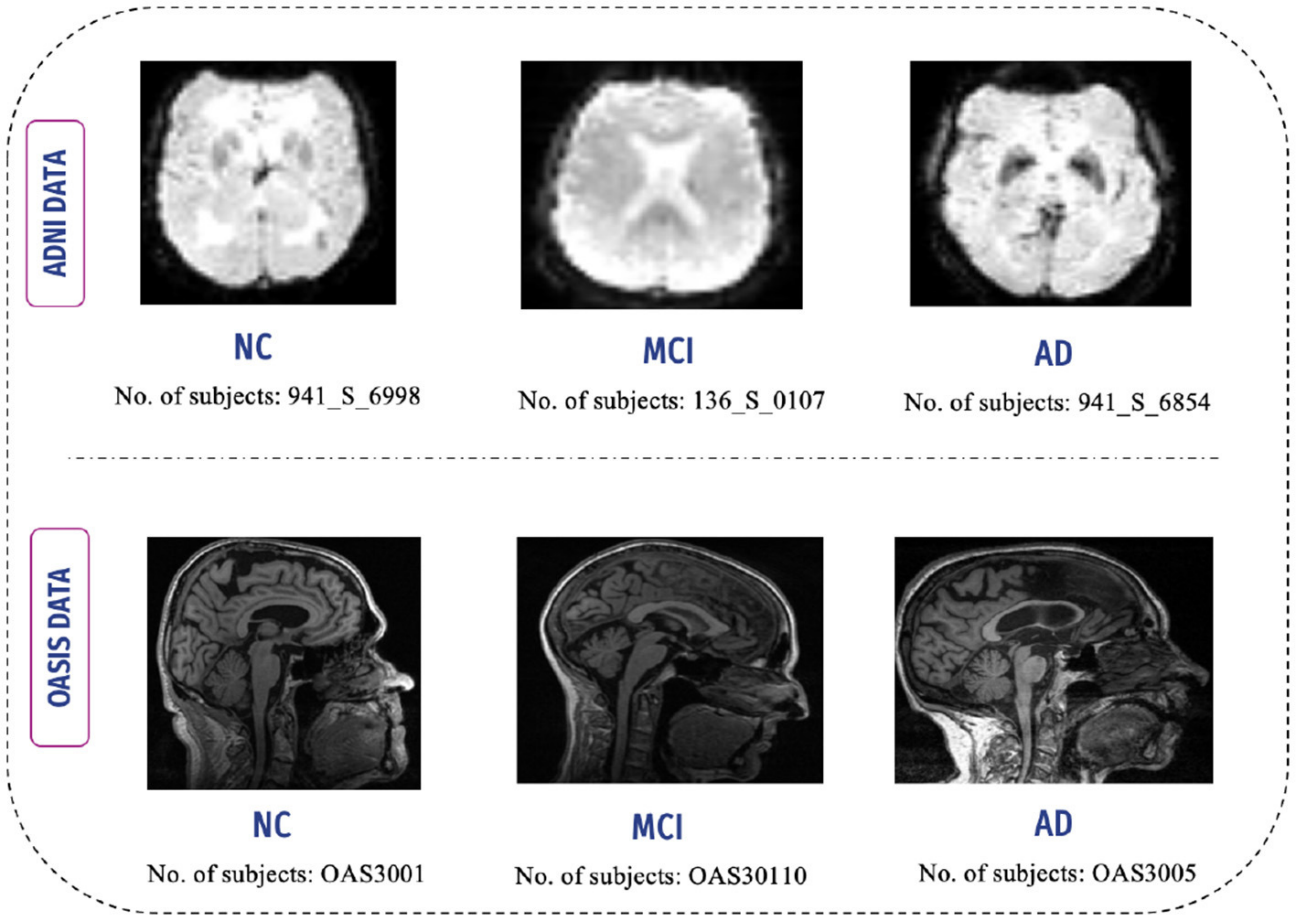
fMRI scans of NC, MCI, and AD subjects.

For the OASIS dataset, fMRI data were accessed through the official website using verified credentials, which allowed free access to the data. Each 3D image is structured as a volume composed of multiple 2D images. The dataset also includes male and female participants across Alzheimer's stages, with 101 individuals (72 males and 29 females) for the AD stage, 95 individuals (40 males and 55 females) for MCI, and 102 individuals (60 males and 42 females) for NC. These datasets collectively provide a robust foundation for exploring Alzheimer's biomarkers and functional connectivity analysis (29). Figure 4 shows the distribution of subjects across the AD, MCI, and NC stages for the ADNI and OASIS datasets.

### 4.2 Preprocessing of fMRI

Since medical images are complex and, hence, hard to extract the features from an image, we need to process images in the dataset using many techniques. All pre-processing techniques are essential for the model during training to make the images clearer, more useful, and perform well. Also, it's necessary during deployment as a part of the model to make the input shape of the images match the input shape of activations from the same brain regions that are accurately captured (30).

Data preparation was conducted using the CONN toolbox (31). The CONN includes several tasks, including segmenting the functional fMRI data, realigning, normalizing, and slicing time correction. It also eliminates noise via de-nosing procedures (32).

#### 4.2.1 Realignment

**Figure 5:**
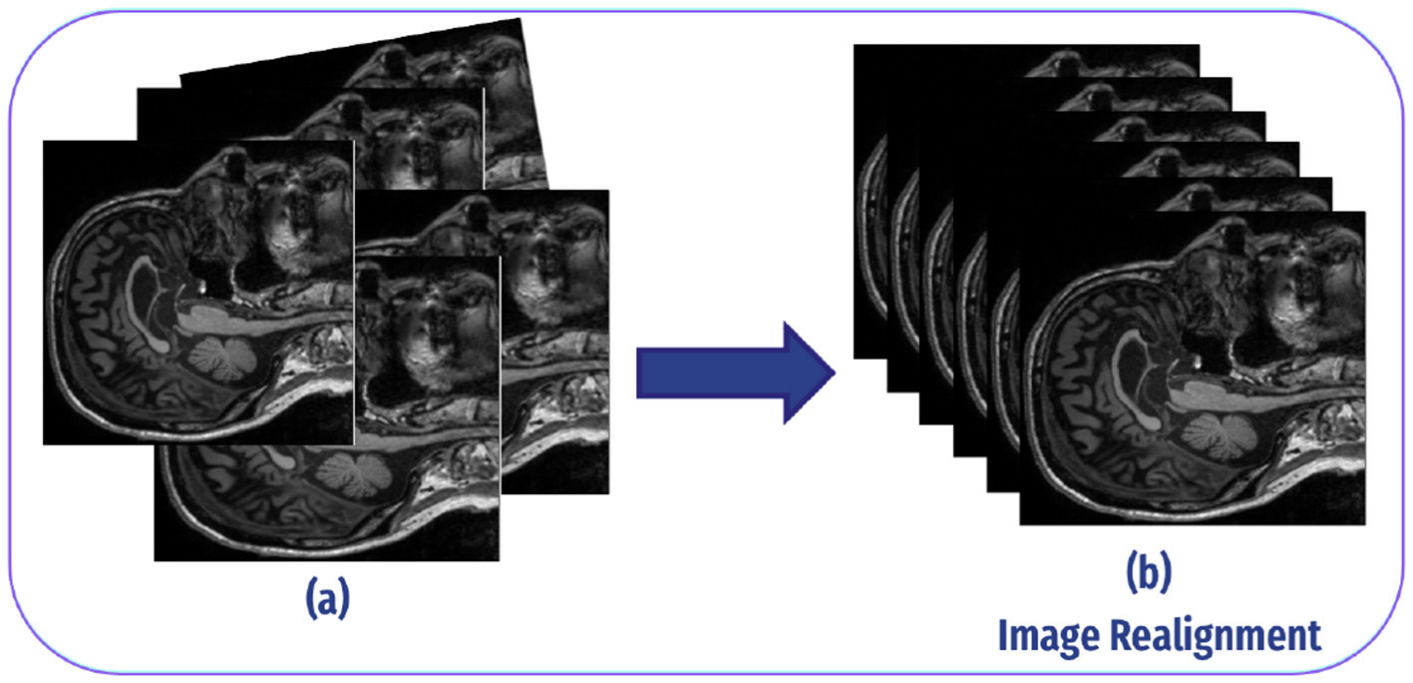
Image realignment: **(A)** Each fMRI scan can differ slightly in angle or position because of minor head movements or inconsistencies in the scanner. As a result, the voxels (3D pixels) in each scan may represent slightly different spatial locations in the brain. This misalignment can create inconsistencies when analyzing data from multiple scans, as the same brain region may not appear consistently in the same voxel at different time points. **(B)** The realignment process modifies each scan to ensure a consistent angle and position, aligning them so that each voxel corresponds to the same spatial location in the brain across all scans.

The second step in our preprocessing pipeline was realignment, aimed at correcting head motion and ensuring that the position of the brain is consistent across all images. This process aligns the functional images so that activations from the same brain regions are accurately captured. In our study, we followed the rational ranges of motion, which were –0.2 to 0.5 for normal controls and –0.2 to –0.15 for individuals with AD (33). As demonstrated in Figure 5.

#### 4.2.2 Registration

**Figure 6:**
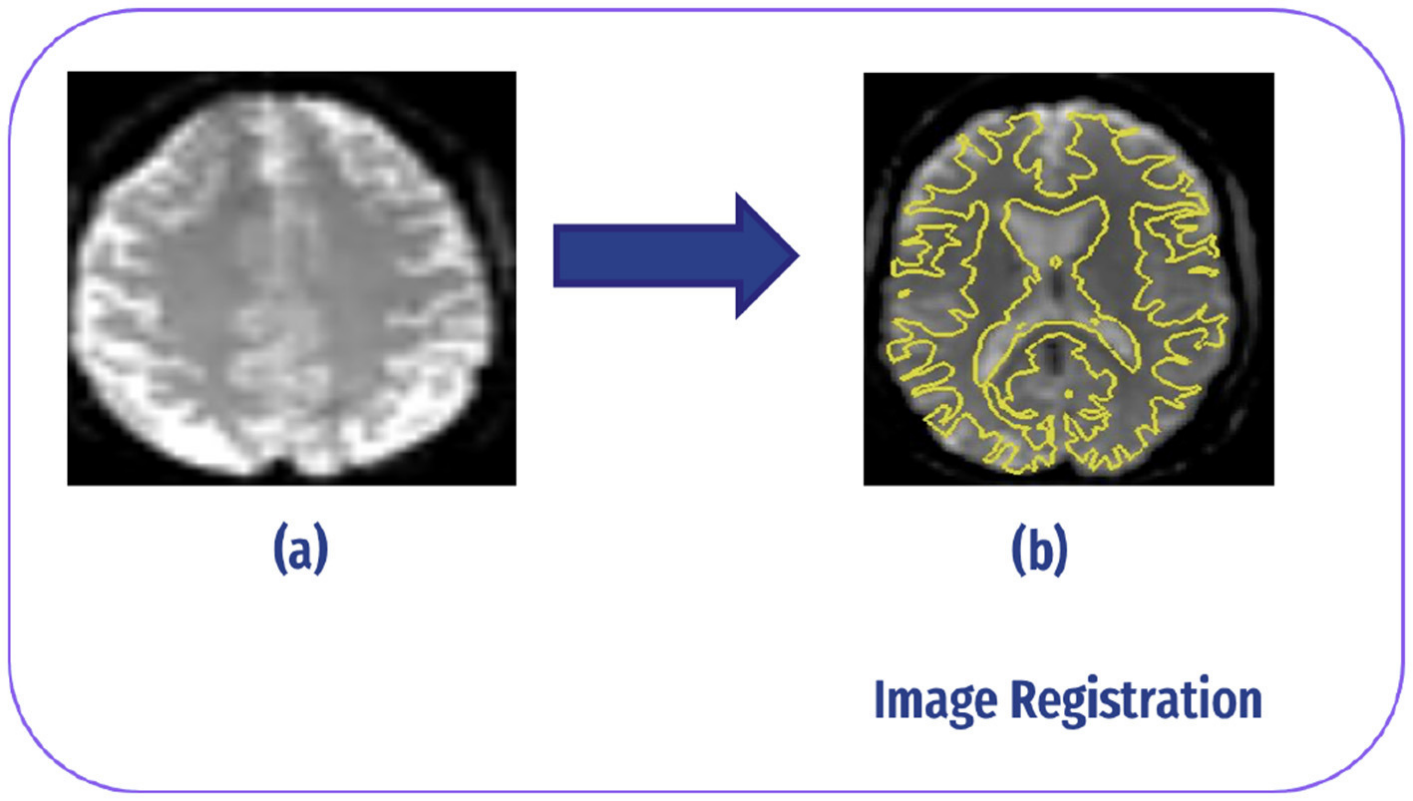
Image registration: **(A)** slice without registration (original image). **(B)** After registrar fMRI with structural MRI.

Image registration in fMRI refers to the process of aligning multiple images acquired from different time points or subjects to a common reference space. This alignment is crucial for combining or comparing data across different scans or individuals. In fMRI studies, image registration typically involves aligning functional images acquired during the scanning session to a standard anatomical template or to each other (34), as demonstrated in Figure 6.

#### 4.2.3 Slice time correction

**Figure 7:**
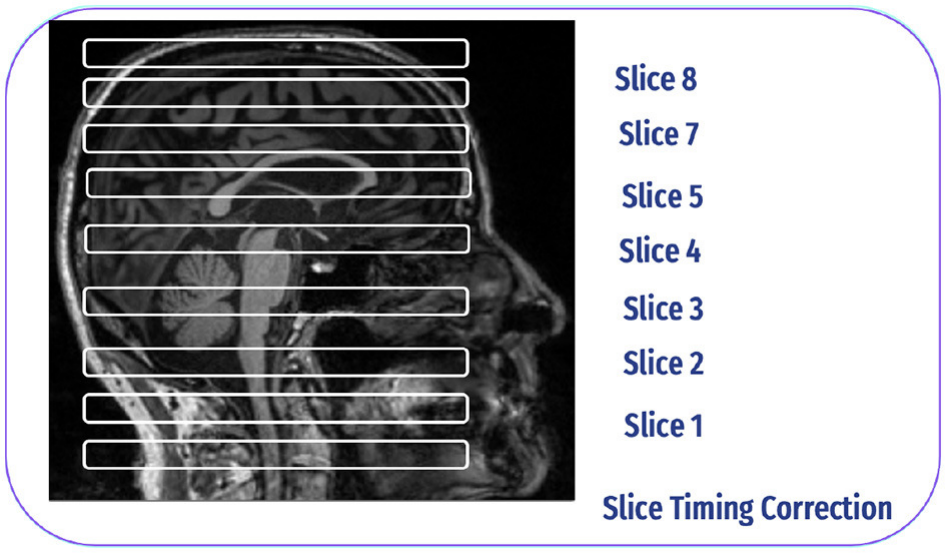
Slice time correction inspired by Poldrack et al. (30).

The ascending method is commonly employed in slice-time correction to address the temporal misalignment of slices in fMRI data. The slices are obtained interleaved rather than sequentially during the collection of fMRI data. The processing starts with inferior slices and progresses to superior slices for each volume for example, in the Philips slice order, the slices are acquired in 1, 4, 7, 6, 9, etc. Slice time correction reorders these slices to be sequential, which is crucial for proper analysis and interpretation of the data (35), as shown in Figure 7.

#### 4.2.4 Spatial normalization

**Figure 8:**
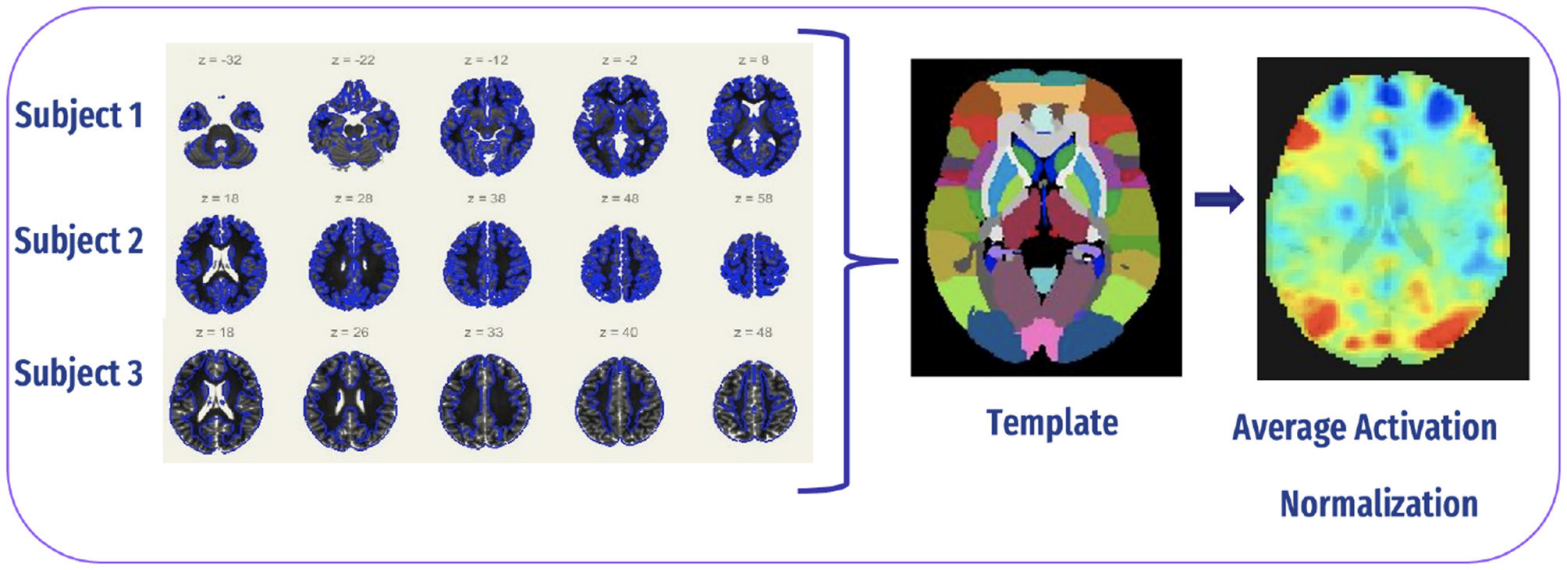
Spatial normalization.

SN is a crucial preprocessing step, particularly because brain sizes can vary significantly among individuals. The goal of this procedure is to reduce the total squared deviations between the functional image and a template image that is commonly used. Normalization matches each brain scan to a common standard space, such the Montreal Neurological Institute (MNI), enabling meaningful comparisons across different people and studies. In our study, we employed indirect normalization to MNI space, ensuring that our functional images were successfully transformed into a standardized space for further analysis (36). The normalization procedure is shown in Figure 8.

#### 4.2.5 Spatial smoothing

**Figure 9:**
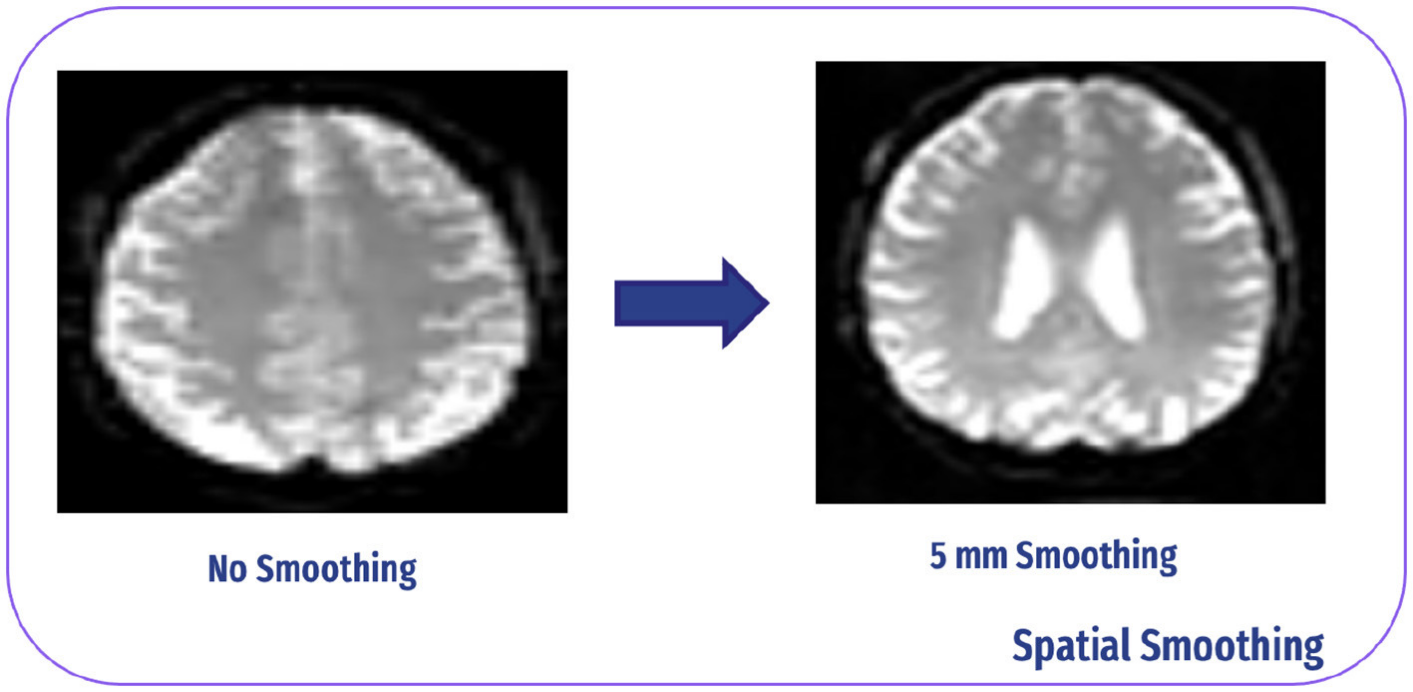
Spatial smoothing.

SS is a crucial preprocessing step in neuroimaging data analysis that raises the signal-to-noise ratio and signal sensitivity. We used a 6 × 6 × 6 mm Gaussian kernel to process the data in our investigation. This kernel size represents the spatial extent over which neighboring voxel values are averaged. By smoothing the data in this manner, we effectively reduce noise and enhance the detect-ability of true underlying signals, improving the quality of subsequent analyses (37). The Smoothing procedure is shown in Figure 9.

### 4.3 Feature engineering

Feature engineering is vital in neuroimaging analysis, particularly for AD and other neurological disorders. This process entails selecting and transforming raw neuroimaging data into relevant features that enhance both model performance and interpretability (38).

#### 4.3.1 Brain regions of interest

**Figure 10:**
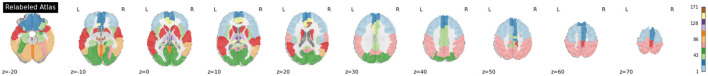
Regions of interest from the AAL3 atlas.

ROIs are specific brain areas that are selected for analysis based on their known or suspected involvement in a particular process or function. In the context of major brain networks, ROIs are typically selected to represent critical nodes or hubs within these networks. In our paper, we utilized automated anatomical atlas 3 (AAL3) for feature extraction to identify relevant brain regions or patterns in the fMRI data. Following this, extract the time series within each ROI (39). In Figure 10 plotting regions of interest from the AAL3 atlas that includes 170 nodes (i.e., regions). Table 2 represents all brain regions in the AAL3 atlas.

**Table 2:** Name of regions for AAL3 atlas.

**Part of regions**		**Name of brain regions**		
Frontal(Front) Lobe	Precentral (LR)	Front Sup (LR)	Front Mid (LR)
	Front Inf Oper (LR)	Front Inf Tri(LR)	Front Inf Orb 2(LR)
	Rolandic Oper (LR)	Supp Motor Area (LR)	Front Sup Medial (LR)
	Front Med Orb (LR)	Rectus (LR)	OFCmed (LR)
	OFCant (LR)	OFCpost (LR)	OFClat (LR)
	Cingulate Mid (LR)	Cingulate Post (LR)	
Temporal Lobe	Olfactory (LR)	Hippocampus (LR)	ParaHippocampal (LR)
	Amygdala(LR)	Heschl(LR)	Temporal Sup (LR)
	Temporal Pole Sup (LR)	Temporal Mid (LR)	Temporal Pole Mid (LR)
		Temporal Inf (LR)	
Occipital Lobe	Calcarine (LR)	Cuneus (LR)	Lingual(LR)
	Occipital Sup (LR)	Occipital Mid (LR)	Occipital Inf (LR)
		Fusiform (LR)	
Parietal Lobe	Postcentral (LR)	Parietal Sup (LR)	Parietal Inf (LR)
	SupraMarginal (LR)	Angular(LR)	Precuneus (LR)
		Paracentral Lobule (LR)	
Cerebellum (Cere)	Cere_Crus1 (LR)	Cere_Crus2 (LR)	Cere 3 (LR)
	Cere4 5 (LR)	Cere 6 (LR)	Cere 7b (LR)
	Cere 8 (LR)	Cere_9 (LR)	Cere 10 (LR)
Vermis (Verm)	Verm 1_2	Verm_3	Verm_4_5
	Verm_6	Verm_7	Verm_8
	Verm_9	Verm_10	
Thalmus	Thal_AV (LR)	Thal_LP (LR)	Thal_VA (LR)
	Thal_VL (LR)	Thal_VPL (LR)	Thal_IL (LR)
	Thal_Re (LR)	Thal_MDm (LR)	Thal_MDl (LR)
	Thal_LGN (LR)	Thal_MGN (LR)	Thal_PuA (LR)
	Thal_PuM (LR)	Thal_PuL (LR)	Thal_PuI (LR)
Insula		Insula (LR)	
Caudate		Caudate (LR)	
Not in Lobe1 wise regain	ACC sup (LR)	N_Acc (LR)	ACC_sub (LR)
	ACC_pre (LR)	N LC (LR)	Raphe_M
	SN_pc (LR)	SN_pr (LR)	VTA (LR)
	SN_pr (LR)	Red_N (LR)	Raphe_D
Not in Lobe2 wise regain	Pallidum (LR)	Putamen (LR)

### 4.4 Functional connectivity

Multi-voxel pattern analysis (MVPA) is a neuroimaging technique that examines activity patterns across multiple brain voxels (volumetric pixels). Unlike traditional methods that focus on average activity within specific brain regions, MVPA analyzes the distribution of activity across voxels to decode or classify cognitive states, stimuli, or tasks. This approach offers a significant advantage over single-voxel analyses, as it provides greater sensitivity for identifying specific cognitive states in the brain. By considering the relationships and interactions between voxels, MVPA can detect subtle and distributed patterns of neural activation that might be missed by region-based analysis. This makes MVPA particularly effective in revealing complex brain processes, offering insights into how different brain areas work together to support cognitive functions. Additionally, MVPA has been widely used in studies involving visual perception, language processing, decision-making, and various other cognitive tasks, contributing to a deeper understanding of brain organization and function (40).

**Figure 11:**
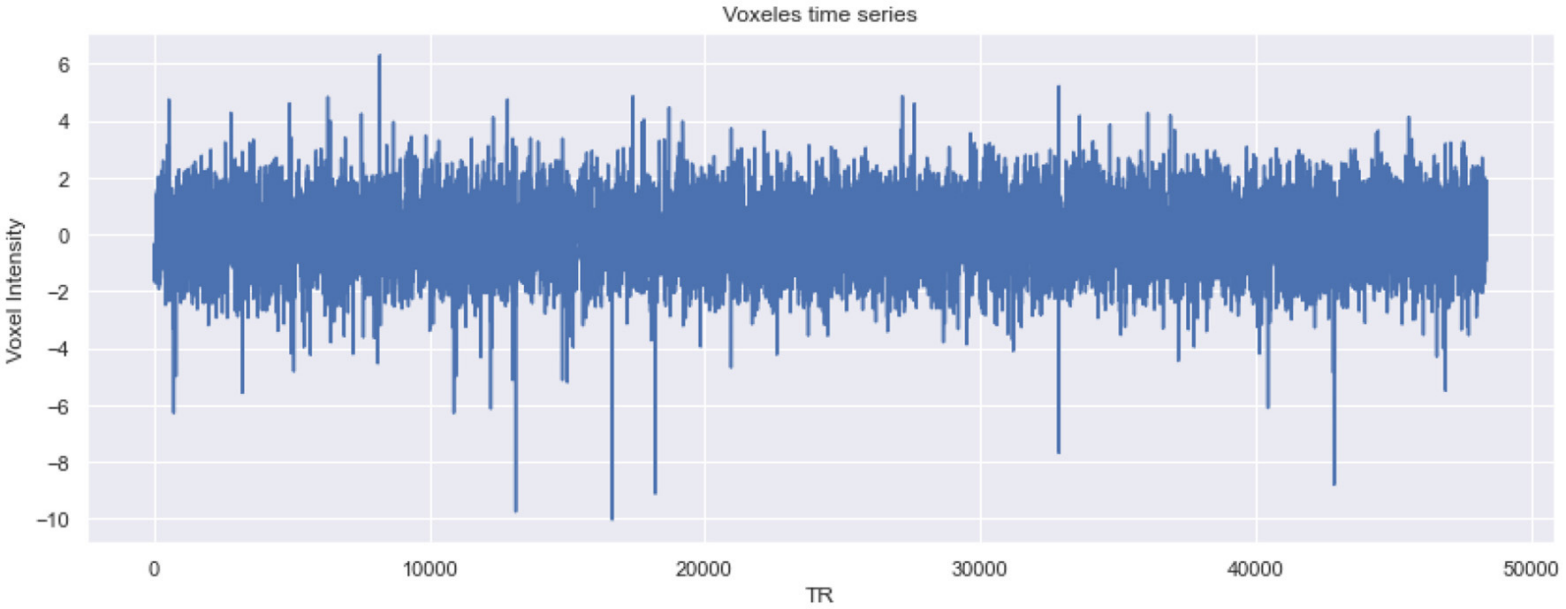
Plot of voxel intensity over time in fMRI analysis.

Figure 11 presents the time series data for a specific voxel within an fMRI dataset, highlighting the changes in intensity over time. The plot shows the intensity values of the selected voxel at various time points. The x-axis is labeled “TR” (time repetitions), while the y-axis is labeled “Voxel Intensity.”

**Figure 12:**
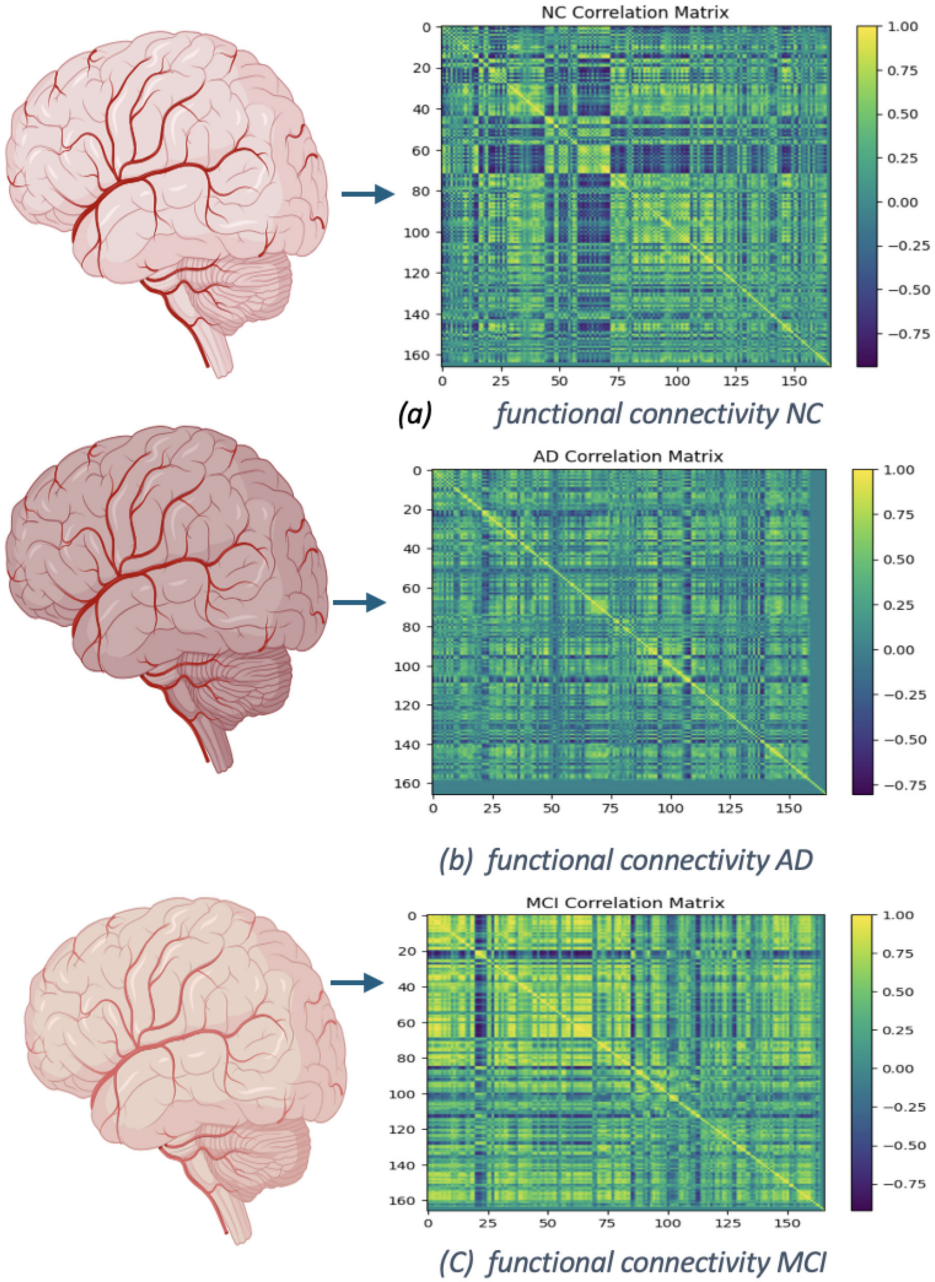
Functional networks of OASIS dataset: **(A)** functional connectivity of NC, No. of Subject 002_S_0295. **(B)** Connectivity of AD, No. of Subject 002_S_0413. **(C)** Functional connectivity of MCI, No. of Subject 002_S_0729.

**Figure 13:**
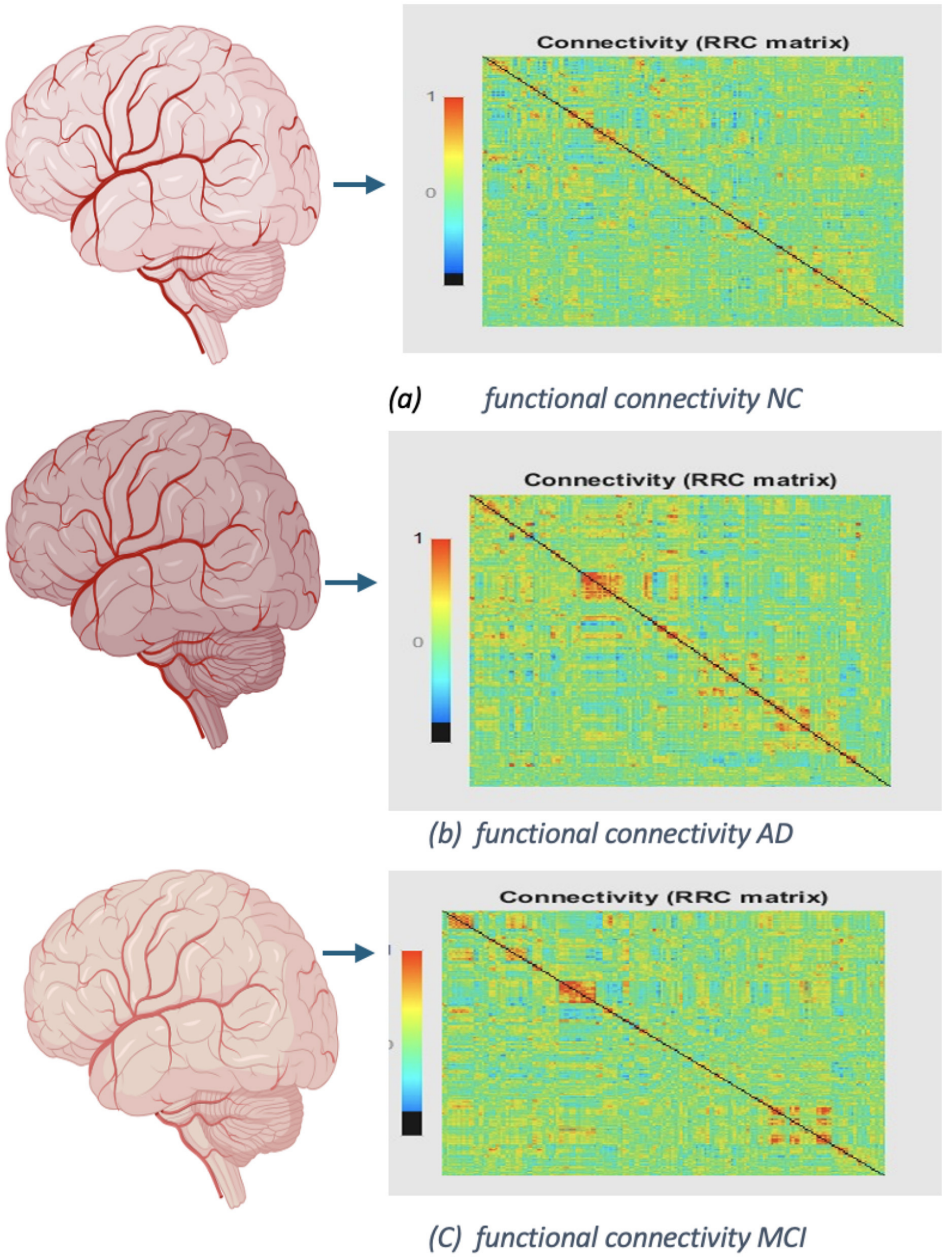
Functional networks of ADNI dataset: **(A)** functional connectivity of NC, No. of Subject OAS30001. **(B)** Connectivity of AD, No. of Subject OAS30090. **(C)** Functional connectivity of MCI, No. of Subject OAS30009.

In Figures 12, 13 display the dimensions of the correlation matrix, which is the number of ROIs squared. This shows how each pair of ROIs for two distinct datasets is connected to the other.

### 4.5 Classifier

**Figure 14:**
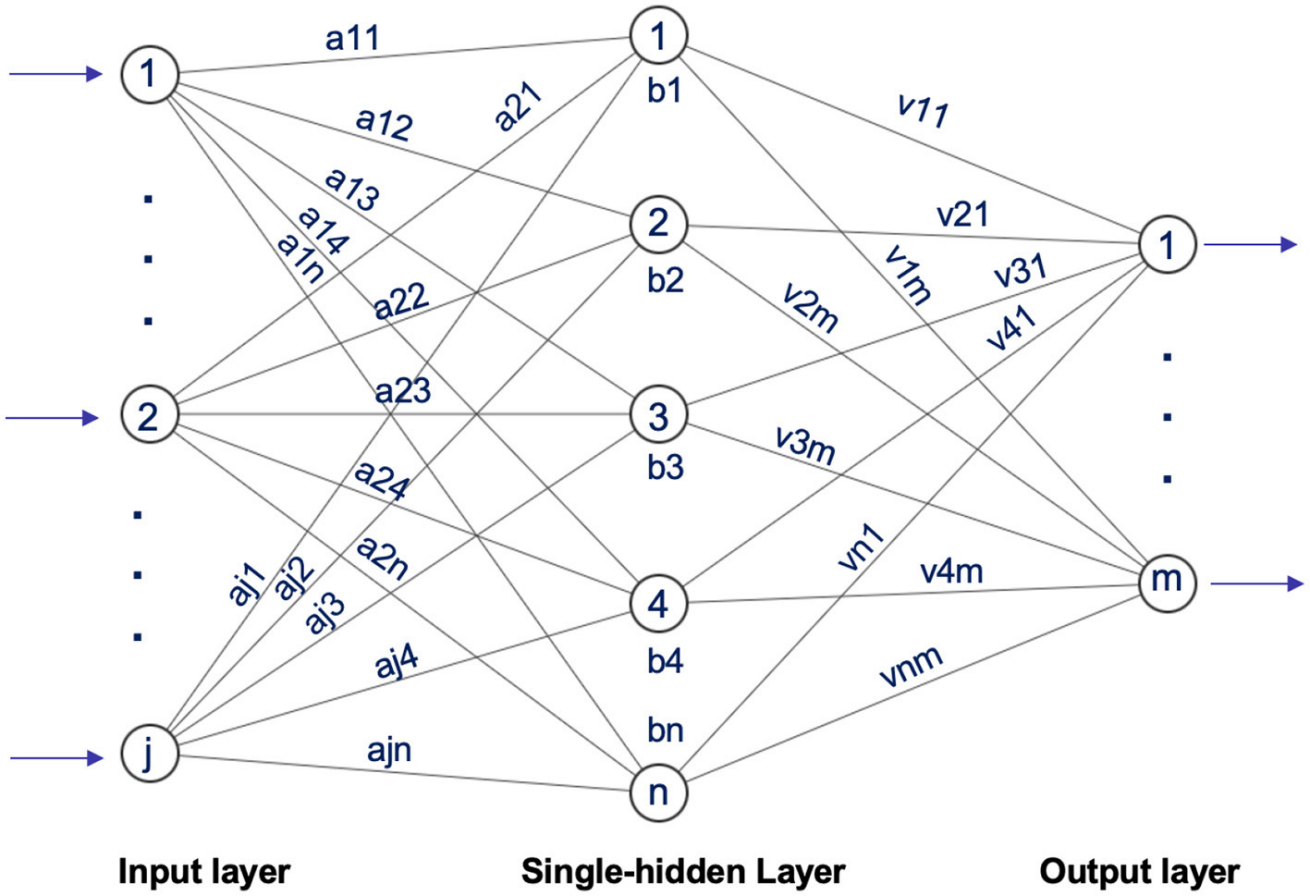
Architecture of extreme learning machine inspired by Ding et al. (43).

In 2004, Huang et al. (41) introduced the Extreme Learning Machine (ELM), a straightforward yet highly effective single hidden-layer feedforward neural network approach. ELM randomly selects input weights and hidden layer biases, calculates output weights analytically, and overcomes the limitations of conventional single-hidden-layer feedforward neural network (SLFN) learning methods. This technique has found extensive applications across diverse disciplines, including illness diagnosis, traffic sign detection, picture quality evaluation, and beyond (42), as shown in Figure 14.

Our proposed model leverages the inherent speed and efficiency of ELM by integrating additional techniques that improve performance on high-dimensional neuroimaging data, especially functional MRI (fMRI). We tackle the complexity of this data using innovative ensemble methods combined with ELM. In this approach, multiple ELM classifiers are trained on various subsets of features or data splits, and their predictions are aggregated. This ensemble method enhances robustness and minimizes the impact of data variability, ultimately boosting classification accuracy. For our Model, we set the parameters as follows: hidden units = 32, activation function = “softmax,” random type = “normal,” and C = 0.1.

### 4.6 Result and performance validation

The proposed AD classification model is evaluated using multiple metrics, each offering distinct insights into performance. Accuracy measures the ratio of correctly predicted positive and negative cases to the total samples, providing an overall assessment of the model's categorization ability as per (44, 45). However, it may be unreliable for imbalanced datasets as mentioned by Alqahtani et al. (46). To address this limitation as suggest by Pruthviraja et al. (47), Salehi et al. (48), and (49) precision, and recall are also used. Precision evaluates the model's ability to correctly identify positive cases among all predicted positives (50), focusing on prediction accuracy (51). Recall measures the model's sensitivity by identifying all positive cases in the dataset (52). The F1-score, which calculates the harmonic mean of precision and recall, balances these metrics, ensuring a comprehensive evaluation of the model's performance (53).

In Table 3 binary classification tasks, the ELM model demonstrated strong performance, achieving an accuracy rate of 87.80% in distinguishing between NC and MCI, 86.16% between AD/NC, 85.9% MCl/NC, and 83.1% AD/MCL/NC. Also, in term of recall we got 87.10% distinguishing between NC and MCI, 86.3% between AD/NC, 85.9% MCl/NC, 83.7% for multi-class classification (AD/MCL/NC). In term of prec we got 87.3% distinguishing between NC and MCI, 86.57% between AD/NC, 85.8% MCl/NC, 89% for multi-class classification (AD/MCL/NC). In term of f1-score we got 87.90% distinguishing between NC and MCI, 86.22% between AD/NC, 85.9% MCl/NC, 83.20% for AD/MCL/NC for OASIS dataset. In ADNI dataset the binary classification tasks demonstrate the strong performance of the ELM model. It achieved an accuracy of 94% in distinguishing between AD and MCI, 93.9% between AD and NC, 90.6% between MCI and NC, and 94.1% for multi-class classification (AD/MCI/NC). In terms of recall, the model achieved 94% for AD vs. MCI, 93.9% for AD vs. NC, 90.68% for MCI vs. NC, and 94% for multi-class classification. Precision results showed 94.82% for AD vs. MCI, 93.8% for AD vs. NC, 92.9% for MCI vs. NC, and 94.04% for the multi-class task. Finally, the F1-score performance was 94.14% for AD vs. MCI, 93.9% for AD vs. NC, 91.3% for MCI vs. NC, and 94.5% for multi-class classification (AD/MCI/NC). SO, the result of ADNI dataset outperform OASIS.

**Table 3:** Comparison of results from different fMRI scan methods with our work.

				**Type of class**				
**References**	**Perf**	**No. of dataset**	**Model**	**AD/MCI**	**AD/NC**	**MCI/NC**	**AD/MCI/NC**
(8)	Acc	-	-	-	-	80.3%	-
	Rec	OASIS	GCN	-	-	-	-
	Prec			-	-	-	-
	f1-Sc			-	-	-	-
(9)	Acc			-	-	-	96%
	Rec	ADNI	-	-		-	96%
	Prec		KNN	-	-	-	97%
	f1-Sc			-	-	-	96%
(11)	Acc			-	-	92%	-
	Rec	ADNI	GCL	-	-	89%	-
	Prec			-	-	95%	-
	f1-Sc			-	-	91%	-
(12)	Acc			-	94.5%	-	-
	Rec	ADNI	CapsNet	-	-	-	-
	Prec			-	-	-	-
	f1-Sc			-	-	-	-
(14)	Acc			-	-	-	97.7%
	Rec	ADNI	CNN	-	-	-	91.3%
	Prec			-	-	-	93.64%
	f1-Sc			-	-	-	-
(15)	Acc			-	-	-	96.7%
	Rec	ADNI	CNN	-	-	-	-
	Prec			-	-	-	97%
	f1-Sc			-	-	-	-
(16)	Acc			-	96.80%	-	-
	Rec	ADNI	SVM	-	-	-	-
	Prec			-	-	-	-
	f1-Sc			-	-	-	-
(18)	Acc			95.47%	95.11%	93.5%	92%
	Rec	ADNI & OASIS	HML	95.47%	95.11%	93.49%	93%
	Prec			96.22%	95.83%	93.34%	92%
	f1-Sc			95.50%	95.13%	93.54%	92%
^*^Our study	Acc			87.80%	86.16%	85.9%	83.1%
	Rec	OASIS		87.10%	86.3%	85.9%	83.7%
	Prec		ELM	87.3%	86.57%	85.8%	89%
	f1-Sc			87.90%	86.22%	85.9%	83.20%
	Acc			94%	93.9%	90.6%	94.1%
	Rec	ADNI		94%	93.9%	90.68%	94%
	Prec			94.82%	93.8%	92.9%	94.04%
	f1-Sc			94.14%	93.9%	91.3%	94.5%

^*^ We measure the performance of our trained model using evaluation metrics.

Comparing the OASIS and ADNI datasets, the ELM model performs significantly better on the ADNI dataset across all metrics. This suggests the ELM model has a higher generalization capacity and may be more compatible with the ADNI data. These findings imply that the ADNI dataset may offer higher-quality or more discriminative features, which enhances the model's ability to distinguish between different cognitive stages more effectively. The performance differences between the two datasets underscore the importance of selecting the right dataset in machine learning studies focused on AD detection. Furthermore, this indicates that ELM models can leverage specific characteristics of detailed datasets for improved classification results.

### 4.7 Experimentation setup

All experiments are performed on a PC with 3.7 GHz Intel Core i7-8GB RAM, 250 GB SSD. Programming environment includes 64-bit Windows 10 pro as operating system, MATLAB R2019a, Python 3.9.12, and TensorFlow. Neuroimaging analysis and processing were conducted using the CONN and Statistical Parametric Mapping (SPM) toolbox, which provides comprehensive preprocessing pipelines for fMRI data.

## 5 Discussion

It is essential to accurately classify MCI as an intermediate stage because this can help in early detection and timely intervention, potentially delaying the progression of AD. Our model's ability to improve classification accuracy at this stage indicates its potential for clinical applications in monitoring cognitive decline. The integration of neuroimaging data, especially functional MRI (fMRI), is crucial for understanding the neural correlates of MCI and AD. This approach enables the identification of specific patterns of brain activity linked to these conditions.

Accessing medical data from local sources is essential for advancing healthcare research and improving patient outcomes. We faced significant challenges in collecting data from local hospitals, mainly due to the scarcity of available local fMRI datasets related to AD. Strict patient privacy regulations and personal concerns about data sharing created obstacles to obtaining the necessary information. While these regulations are vital for protecting patient confidentiality, they can also impede research progress by limiting the availability of relevant datasets.

Changes because of AD, About 100 billion neurons with widespread, growing delays make an adult brain in good health. Individual neurons can connect to other neurons thanks to their expansions. Information is transferred at these interconnections, known as synapses, in the form of brief chemical outbursts emitted by one neuron and picked up by a sensory cell. About 100 of these are further harmed by the brain's diminished capacity so that its primary energy, glucose, is metabolized.

According to studies, the brain alterations linked to Alzheimer's may start 20 years or earlier symptoms manifest. In the early stages of brain alterations, the brain compensates for these changes, enabling individuals to continue functioning normally. However, as neuronal damage progresses, the brain's ability to compensate diminishes, leading to mild cognitive decline. Over time, this decline can interfere with daily activities and impact memory and problem-solving abilities. Recognizing these early signs is crucial for timely intervention and management. Eventually, neuronal loss is so severe that people exhibit overt cognitive decline, comprising signs like memory loss or time- and place-related confusion. Even later, basic physical processes like swallowing become compromised. Although some of the early brain changes related to AD can be recognized in study designs, further study is required to improve the accuracy of the instruments before they can be used in clinical settings. Additionally, although several are being evaluated in clinical studies, there are now no medications that can prevent, slow, or stop these alterations (54)

## 6 Conclusions

The degenerative and irreversible brain condition known as AD progressively impairs thinking and memory abilities, eventually resulting in the loss of mental function. In this paper, we computed functional connectivity (FC) using Multivariate Pattern Analysis (MVPA). Then we classified fMRI data from the longitudinal OASIS-3 and ADNI datasets into binary and multi-class categories using the Extreme Learning Machine (ELM) model. While our approach demonstrates promising results, several limitations need to be considered. The use of deep learning models, such as the Extreme Learning Machine (ELM), although efficient, introduces challenges. Deep learning models are often viewed as “black boxes,” making it difficult to interpret the features they learn and understand the decision-making process. This lack of interpretability is a significant limitation, especially in clinical settings, where understanding the rationale behind predictions is essential. Future research will employ our techniques to analyze electroencephalography (EEG) recordings of individuals in the resting state (i.e., with their eyes closed) who are suffering from AD, front temporal dementia, and NC (55). We are going to use EEGNet, a specific kind of neural network architecture made to handle EEG data. Using unprocessed fMRI data, our suggested approaches can accurately predict Alzheimer's illness. This research can improve our understanding of the disease and how it progresses, which has enormous clinical relevance.

## Data Availability

The original contributions presented in the study are included in the article/supplementary material, further inquiries can be directed to the corresponding authors.
